# Macular edema after siponimod treatment for multiple sclerosis: a case report and literature review

**DOI:** 10.1186/s12883-023-03333-0

**Published:** 2023-07-31

**Authors:** Qingsheng Li, Li-Jun Jing, Yanfei Li, Yanjie Jia

**Affiliations:** 1 Department of Neurology, The First Affiliated Hospital of Zhengzhou University, Zhengzhou University, Zhengzhou, Henan China; 2 Academy of Medical Sciences, Zhengzhou University, Zhengzhou, Henan China

**Keywords:** Multiple sclerosis, Siponimod, Sphingosine 1-phosphate, Macular edema, Case report

## Abstract

**Background:**

As a modulator of the sphingosine 1-phosphate receptor, siponimod is administered as a therapeutic intervention for multiple sclerosis. A previous phase 3 study first reported siponimod-associated macular edema. Since that report, there were only few relevant reports in clinical settings. Here, we report a case of secondary progressive multiple sclerosis developed macular edema after siponimod treatment. We also review the progress of sphingosine 1-phosphate receptor modulators, elaborate on accepted mechanisms in treating multiple sclerosis, and discuss the causation of siponimod-associated macular edema.

**Case presentation:**

A 38-year-old Chinese female patient with secondary progressive multiple sclerosis, who had recurrent numbness of the limbs and right leg fatigue, developed mild macular edema following 4 months of siponimod treatment. The macular edema resolved after discontinuing the medication, and did not recur after resuming siponimod.

**Conclusion:**

Although siponimod-associated macular edema may be rare, mild, transitory, and manageable, it cannot be ignored and requires ongoing vigilance.

## Background

Multiple sclerosis (MS) is characterized by inflammatory axonal demyelination in the central nervous system with autoimmune dysfunction that typically results in damage to the brain, brainstem, optic nerve(s), and spinal cord. It is the most common nontraumatic cause of neurologic disability in young adults (age < 40 years). The estimated global prevalence of MS is 5–300 per 100,000 persons, with increased incidence reported in higher-latitude locations [1]. Approximately 85% of patients have relapsing-remitting MS (RRMS) at onset. In RRMS cases, focal lesions and neurologic disabilities flare up, nearly or completely recover, and repeatedly relapse, leading to increasingly severe discomfort and disability over time. In more than 50% of cases, RRMS becomes secondary progressive MS (SPMS) with progressive inflammation and ongoing neurodegeneration [1].

It is important to identify the specific subtypes of MS via the time course of the initial symptoms and disease evolution to ensure the selection of appropriate disease-modifying therapies (DMTs). Some DMTs efficiently prolong the interval between the first clinical event and relapse with efficacy rates of 29–68%, including sphingosine 1-phosphate (S1P) receptor (S1PR) modulators. Siponimod is a new-generation selective S1PR modulator for S1PR 1 and 5 [2]. The BOLD study, a randomized, double-blind phase 2 clinical trial that evaluated siponimod treatment for adults with RRMS, showed that siponimod treatment could improve clinical outcomes and reduce lesional severity compared with placebo [3, 4]. The EXPAND study, a clinical multicenter phase 3 trial, in which titration was set to 2 mg siponimod to avoid chronotropic cardiac effects, showed that siponimod had significant positive effects and acceptable safety in patients with SPMS [5].

The previous BOLD study and its extension reported the absence of macular edema (ME) [3, 4]. The EXPAND study first reported that siponimod resulted in increased incidence of ME compared with placebo [5]. Following the EXPAND study, there were few reported cases of ME after siponimod administration. Herein, we describe the case of a patient with MS who developed ME after siponimod treatment and review the mechanisms of S1PR modulators for both clinical effects and ME occurrence.

## Case presentation

In September 2006, a 23-year-old Chinese female patient was admitted to the Department of Neurology with numbness below the chest, lower limb weakness, constipation, and urinary retention for 1 week after catching a self-reported cold. Prior to this incident, the patient had no history of neurologic or autoimmune diseases. The patient’s family had no history of any genetic disorders. Physical examination revealed hypoalgesia below the T2 level, grade 4 muscular strength of the lower limbs, brisk tendon reflexes in both lower extremities, and bilateral positive pathological reflex. Spinal magnetic resonance imaging (MRI) revealed abnormal lesions extending from C3 to T9. The patient was initially diagnosed with acute myelitis. RRMS was confirmed after symptom relapse occurred twice in 9 months, along with the development of weakness and numbness of all limbs. These symptoms remitted after high-dose intravenous methylprednisolone pulse therapy. Upon MRI re-examination, abnormally long T1 and T2 signals at levels from C3 to C5 were observed. After discharge, the patient received oral prednisone; the dose was tapered over the course of 6 months. Following treatment, the patient maintained a stable condition for years.

**Fig. 1:**
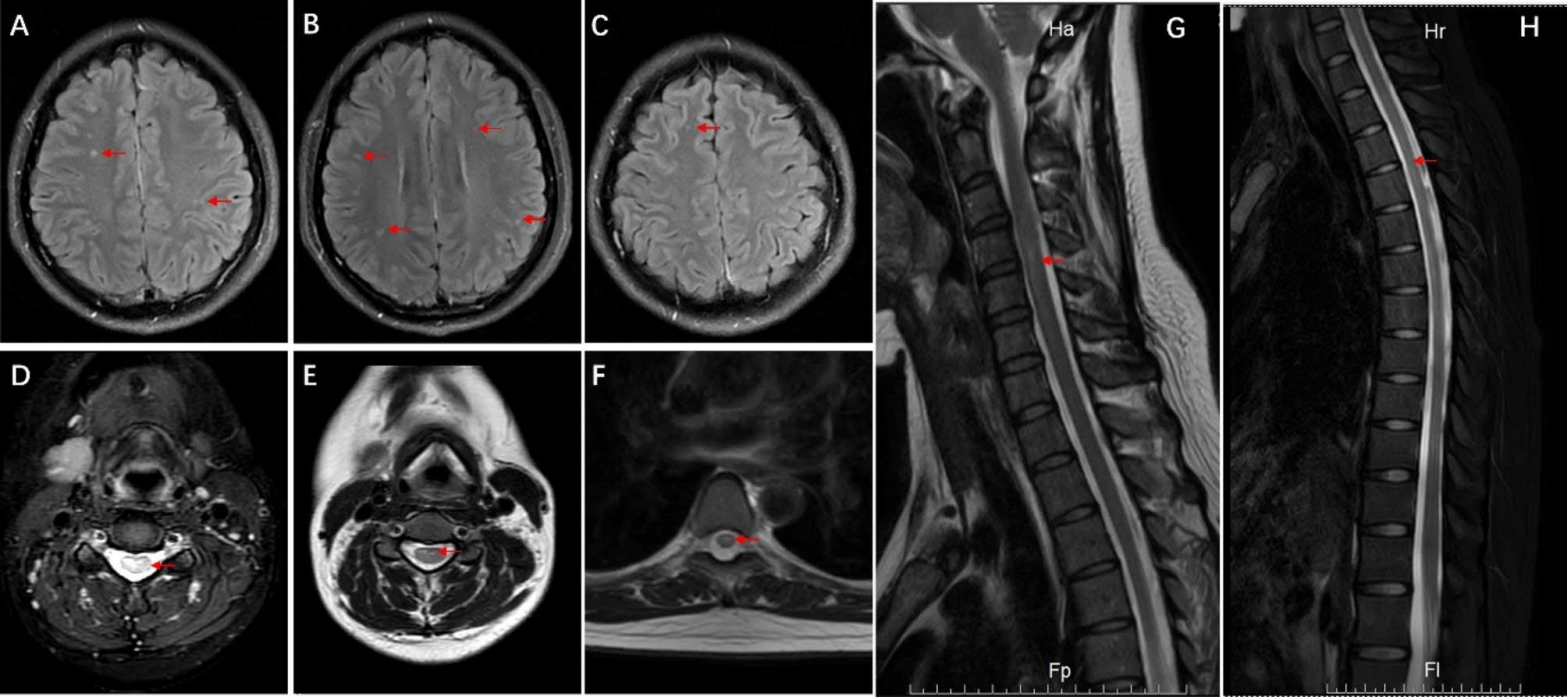
Brain MRI (T2-FLAIR) performed in November 2021 shows multiple hyperintense white matter lesions adjacent to the bilateral ventricle, at the bilateral frontal lobe, and at the right centrum semiovale and venous developmental anomaly at the left frontal lobe (**A–C**). DWI shows no abnormalities. Axial cervical (T2) and thoracic (T2-FS) spinal MRI present multiple hyperintense patchy lesions along the cervical and thoracic segments (**D–F**). Sagittal MRI (T2–FS) shows banded lesions along the thoracic and cervical cord at the C4–C6 and T2–T9 levels (**G, H**). Strong signals are not present on enhancing MRI. Red arrowheads indicate the lesions. DWI, diffusion-weighted imaging; MRI, magnetic resonance imaging; T2, T2-weighted imaging; T2-FLAIR, T2-weighted fluid-attenuated inversion recovery imaging; T2-FS, fat-saturated T2-weighted imaging

In November 2021, the patient was admitted to our hospital because of recurring numbness of the limbs and fatigue in the right leg after an upper respiratory tract infection. Physical examination revealed normal vision, visual field, ocular fundus, muscle strength and tone, normal superficial and deep sensations, and hyperreflexia of the bilateral knee and right ankle. MRI revealed multiple demyelinating lesions in the brain, thoracic cord, and cervical cord (Fig. 1). Aquaporin 4, glial fibrillary acidic protein, myelin oligodendrocyte glycoprotein, and myelin basic protein were negative in both plasma and cerebrospinal fluid (CSF). CSF pressure was 230 mmH_2_O, and CSF cytological, biochemical, immunological, microbiological, and virological tests were normal. The patient was diagnosed with MS, and the current symptoms indicated another RRMS attack. The diagnosis was based on the following: the patient was a young adult woman with a history of acute-onset MS and MRI findings showing multiple temporal and spatial attacks; applicable 2017 McDonald’s diagnostic criteria were evident [6]; exclusion of neuromyelitis optica, intracranial infection, genetic leukoencephalopathies, autoimmune encephalomyelitis, and intracranial space-occupying lesions was confirmed. After methylprednisolone pulse therapy, the patient was discharged from the hospital with symptomatic relief. Neurologists advised that the patient should be given DMT drugs to reduce the risk of relapse.

**Fig. 2:**
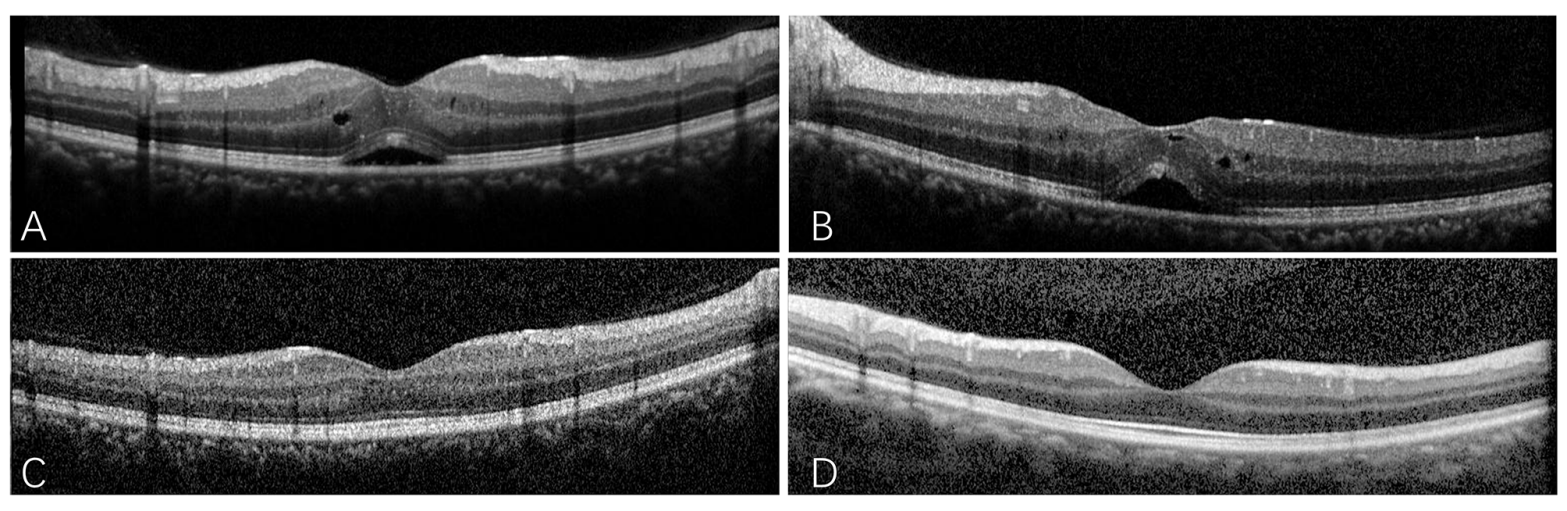
OCT images. The left (**A**) and right eyes (**B**) exhibit mild macular edema after 4 months of siponimod therapy. One month after withdrawal of siponimod, OCT shows resolution of left macular edema (**C**) and alleviation of right eye lesions (**D**). OCT, optical coherence tomography

**Fig. 3:**
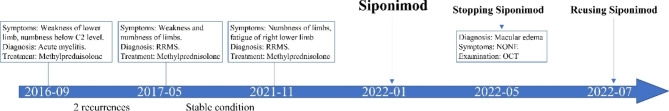
Disease course. OCT, optical coherence tomography; RRMS, relapsing-remitting multiple sclerosis

From January 2022, siponimod was titrated up to 2 mg for 4 days, and this dose was maintained. After 1 month, routine blood analyses showed a mildly reduced lymphocyte count (0.99 × 10^9^/L) and percentage (19.8%). Slight liver dysfunction was observed, alanine aminotransferase [ALT] levels were 52 U/L. The patient continued taking the medication without any discomfort. Following the instructions of an internal medicine physician and based on previous studies [5], the patient was advised to undergo optical coherence tomography after 4 months to evaluate the fundus oculi and macula, which revealed mild ME in both eyes without vision changes (Fig. 2). Blood tests revealed a more extensive decrease in lymphocytes (0.75 × 10^9^/L; 13.4%) and a more severe liver dysfunction (ALT, 66 U/L) than that indicated by previous results. The patient had no history of diabetes or ocular disease. Subsequently, siponimod use was discontinued for 1 months, and ophthalmic inspections showed an improvement in the patient’s ME (Fig. 2). The doctors suggested that the patient take siponimod a second time and be examined by an ophthalmologist once a month. After 1 year of administration, no changes in vision were observed. Figure 3 shows the timeline of the patient’s disease course.

## Discussion and conclusion

We described the case of a female patient diagnosed with RRMS who developed mild ME after siponimod treatment. The patient’s ME improved after stopping siponimod administration and did not recur after resuming siponimod. After the EXPAND study, there were few cases reporting ME as a side effect of siponimod in clinical settings. All previously reported cases were women aged over 50 years, who had severe macular edema that resolved after discontinuing siponimod, but did not resume the medication [7–9]. In contrast, this report describes a case of a 38-year-old woman with SPMS whose mild macular edema also improved after discontinuing siponimod, and she continued to take it without recurrence of macular edema.

ME is often caused by age, diabetes, inappropriate drug usage, preexisting ocular conditions, and other factors, and it results in retinal degeneration and vision loss. The patient in this case had no history of diabetes or ocular diseases. Few studies have reported on the role of MS in microcystic ME, which is a common ocular disease that is rarely caused by severe optic neuropathy and/or optic atrophy (10). Analyzing the present case, the authors differentiated MS-induced microcystic ME from S1PR modulator-induced ME through symptom analysis, MRI, and optical coherence tomography. Many drugs can cause ME, including aminoglycosides, taxanes, niacin, thiazolidinediones, tamoxifen, interferons, and ophthalmic topical medications [11, 12]. However, the only candidate drug administered to our patient was siponimod.

The only approved indication for S1PR modulators is MS. S1PR1, the primary target, regulates lymphocyte reactivity to the S1P chemotactic gradient between lymphoid tissues and lymphocytes. When S1PR modulators block S1PR1 function, lymphocytes stop secretion into the blood from the lymph nodes and thymus, which may lead to decreased inflammatory cell migration and confined cytokine expression in the central nervous system [13]. S1PR1 plays multiple regulatory functions, such as modulating vascular development, tone and permeability, neurogenesis, bronchial tone, hyperreactivity, and lymphocyte trafficking. S1PRs have five subtypes found throughout the body with diverse functions, but their clinical functions and side effects have not been well studied [14]. They are abundantly located in the retinal and ocular posterior segments and are closely associated with ME [15].

However, the mechanisms by which S1PR modulators induce ME remain unclear. S1P enables barrier function reinforcement via S1PR1 in vitro, which may contribute to the inhibition of exudation and choroidal neovascularization—the two characteristics of exudative age-related macular degeneration [15]. S1PR2 and S1PR3 promote chemokines and vascular regeneration, which may damage the retinal pigment epithelium in vitro [16]. Moreover, S1PR2 knockout could cause retinal neovascularization retardation in vivo [15, 17].

Fingolimod is the first approved S1P structural analog with low selectivity. Several studies on the mechanism of fingolimod-associated ME may provide some reference for understanding similar effects exerted by other S1PR modulators [2]. Some studies have inferred that blocking S1PR1 on endothelial cells in retinal vessels may break intercellular and cell-matrix adhesion clusters and increase permeability, leading to edema [18]. However, others believed that fingolimod could activate S1PR1 in the retina and subretinal structures and protect against macular degeneration [15, 19], which is contrary to the hypothesis that these modulators block S1PR1 to protect patients against MS [13]. Several questions have arisen in the literature: does fingolimod activate or block S1PR1 in different organs, and could S1P structural analogs with side effects causing ME treat macular degeneration? S1PR modulators have complicated effects on receptors, acting as traditional agonists or agonists with functional antagonism in some contexts. The prevailing view is that ligand binding leads to internalization and functional antagonism of S1PR. However, evidence suggests that S1PR modulators may initially act as potent agonists and become rapidly transformed into antagonists [20]. These complex interactions dictate that S1PR modulators have complex and often contradictory clinical implications and the potential to treat multiple diseases. Thus, it is important to improve the latent abilities of these receptors by improving receptor and/or tissue selectivity.

Siponimod is a newly developed selective inhibitor of S1PR1 and S1PR5 that aims to decrease the incidence of side effects, such as bradycardia [2]. It can readily penetrate the blood–brain barrier and is distributed in the central nervous system [21]. Its elimination period (7 days) and half-life (30 h) are shorter than those of fingolimod. To a certain extent, a short drug half-life ensures the rapid recovery from side effects after drug cessation [2]. Notably, ME incidence after fingolimod treatment is rare, mild, transitory, and manageable [22]. Some studies have shown that fingolimod-associated ME is treatable without drug cessation [23]. In the EXPAND trial and its extension study, few cases of macular edema were reported, and none of them provided treatment plans or prognosis information [24]. The incidence of siponimod-induced macular edema was greatly increased in patients with diabetes and advanced age [25]. In clinical practice, only a few reports involved siponimod-induced macular edema, and all cases were cured after discontinuing the medication. However, none of the previous cases mentioned continuing siponimod to control SPMS. In this case, the ophthalmologist advised the patient to discontinue siponimod for safety reasons. One month later, the macular edema improved and she returned to the neurology department. Considering the possibility of SPMS worsening, siponimod was the only proven effective drug for SPMS at that time, and the patient’s macular edema was resolved and manageable. Literature review confirmed that there were cases of fingolimod-treated ME without discontinuation, and that siponimod-induced ME was controllable and reversible. Finally, after consulting with the ophthalmologist and weighing the pros and cons, the patient was advised to continue oral siponimod and adhere to ophthalmologic follow-up. The patient has been on DMT treatment for more than a year, with no worsening of neurological symptoms or recurrence of significant drug complications.

There are some limitations to our case report. First, the patient did not undergo an ocular examination before siponimod treatment, as she had no history of ocular diseases. However, the patient’s ME improved after discontinuing DMT, which suggests that the ME was induced by siponimod. Second, the time between the patient restarting siponimod and ME examination was short; hence, The authors will follow-up on the patient’s ongoing progress. The siponimod dose given to the patient was chosen based on the dose recommended by the EXPAND study to reduce the side effects. In this case, the ME was mild, improved quickly after withdrawal of siponimod, and did not result in relapse after using siponimod again in follow-up treatment. It is consistent with the mechanism that S1PR modulators internalize and inhibit the S1PR after the early activation [20]. If this patient’s ME recurs, the researchers will maintain the siponimod treatment and perform ocular consultation and treatment, as reported in a fingolimod case report [23]. This patient’s case may help neurologists and ophthalmologists working with siponimod-associated ME.

In conclusion, the researchers described the case of a young female patient diagnosed with RRMS who developed ME after siponimod treatment. This case suggests that siponimod-associated ME is rare, mild, and manageable; however, regular optic assessment remains crucial to avoid delays in treatment and to promote patients’ quality of life.

## Data Availability

The datasets used and/or analysed during the current study available from the corresponding author on reasonable request.

## List of abbreviations

DMTs: Disease-modifying therapies
ME: Macular edema
MRI: Magnetic resonance imaging
MS: Multiple sclerosis
RRMS: Relapsing-remitting MS
S1P: Sphingosine 1-phosphate
S1PR: Sphingosine 1-phosphate receptor
SPMS: Secondary progressive MS
